# Role of Moonlighting Proteins in Disease: Analyzing the Contribution of Canonical and Moonlighting Functions in Disease Progression

**DOI:** 10.3390/cells12020235

**Published:** 2023-01-05

**Authors:** Mario Huerta, Luis Franco-Serrano, Isaac Amela, Josep Antoni Perez-Pons, Jaume Piñol, Angel Mozo-Villarías, Enrique Querol, Juan Cedano

**Affiliations:** Institut de Biotecnologia i Biomedicina, Departament de Bioquímica i Biologia Molecular, Universitat Autònoma de Barcelona, Cerdanyola del Vallés, 08193 Barcelona, Spain

**Keywords:** moonlighting proteins, wound-healing, cancer wound-healing, stress, inflammation, stem cells, proliferation, fibrosis

## Abstract

The term moonlighting proteins refers to those proteins that present alternative functions performed by a single polypeptide chain acquired throughout evolution (called canonical and moonlighting, respectively). Over 78% of moonlighting proteins are involved in human diseases, 48% are targeted by current drugs, and over 25% of them are involved in the virulence of pathogenic microorganisms. These facts encouraged us to study the link between the functions of moonlighting proteins and disease. We found a large number of moonlighting functions activated by pathological conditions that are highly involved in disease development and progression. The factors that activate some moonlighting functions take place only in pathological conditions, such as specific cellular translocations or changes in protein structure. Some moonlighting functions are involved in disease promotion while others are involved in curbing it. The disease-impairing moonlighting functions attempt to restore the homeostasis, or to reduce the damage linked to the imbalance caused by the disease. The disease-promoting moonlighting functions primarily involve the immune system, mesenchyme cross-talk, or excessive tissue proliferation. We often find moonlighting functions linked to the canonical function in a pathological context. Moonlighting functions are especially coordinated in inflammation and cancer. Wound healing and epithelial to mesenchymal transition are very representative. They involve multiple moonlighting proteins with a different role in each phase of the process, contributing to the current-phase phenotype or promoting a phase switch, mitigating the damage or intensifying the remodeling. All of this implies a new level of complexity in the study of pathology genesis, progression, and treatment. The specific protein function involved in a patient’s progress or that is affected by a drug must be elucidated for the correct treatment of diseases.

## 1. Introduction

Moonlighting proteins refer to those proteins with two or more functions performed by a single polypeptide chain. Moonlighting proteins present alternative functions (named canonical and moonlighting, respectively) which are mostly affected by cellular localization, cell type, oligomeric state, concentration of cellular ligands, substrates, cofactors, products, or post-translational modifications [1,2,3,4]. The canonical function is evolutionarily conservative and fundamental. Rather, the moonlighting functions are different from the canonical ones and are performed at a different location or under unusual conditions. In this way, the moonlighting proteins represent an evolutionary advantage for the cell and the organism, but they pose a drawback for researchers and physicians. The acquisition of a new protein function can become an advantage for cell and organism because this reduces the number of genes to be expressed and the number of proteins to be synthesized. However, these moonlighting proteins complicate the interpretation of knock-outs/knock-ins, DNA arrays, transcriptomics, transcriptomics metabolomics, systems biology, drug pharmacokinetics, pharmacodynamics and toxicity assays. 

There are three multitasking and moonlighting-protein databases: MultitaskDB at http://wallace.uab.es/multitaskII/ [5], MoonProt at http://www.moonlightingproteins.org [6] and MoonDB [7]. In a previous work using proteins from MultitaskProtDB we have identified that 78% of human moonlighting proteins are involved in disease; 48% of the moonlighting proteins are targets of current drugs, and 25% of the moonlighting proteins have a moonlighting function related to the virulence activity of the pathogen [8].

A typical moonlighting protein has a pair of independent autonomous functions, i.e., the enzyme and transcription factor is the most common pair [9]. From the biochemical and physiological point of view, both canonical and moonlighting functions can be equally considered as “standard” functions, and the moonlighting function is usually considered evolutionarily independent of the canonical one. However, in the present work we show a number of examples in which both functions are closely related in clinical conditions, sometimes synergistically, and others antagonistically.

Protein function also has a hierarchic attribute. As we demonstrate in the present work, proteins have one or more functions at cellular/subcellular levels, but they can present other functions at higher levels (tissue and organ) such as being hormone-like, growth factor, etc. For example, the canonical function of chromatin non-histone High Mobility Group Protein B1 (HMG-1) at the cellular level is in a chromatin structure, but it presents other moonlighting functions at higher hierarchic levels: it promotes cell adhesion, activates coagulation, etc. We show many proteins that activate their higher-hierarchy moonlighting functions in response to pathological conditions in the present work that, trigger a more systemic response as a result.

Several authors have reported the link between moonlighting proteins and human diseases [8,10,11,12]. In most cases the involvement in the pathology is due to a malfunction of one of their different functions, caused by mutations, novel interactions, gene up or down-expression, etc. Jeffery has pointed out that sometimes the mutation adds a second pathological function [12] instead of inhibiting the canonical function. This author shows examples related to mutations affecting the conformation or novel interactions. For instance, mutations in the dimer interface of dihydrolipoamide dehydrogenase result in the appearance of protease activity [12].

In the present work we go further and show that, in many cases, the canonical and moonlighting functions of proteins involved in pathology have a dependence between them. We found multiple moonlighting functions exerted by different proteins involved in the response to damage. These moonlighting functions, once activated by specific pathological conditions, can exert a synergistic or opposite regulation of the processes that carry out the damage response. This response to damage may involve a more systemic compromise if the pathological condition cannot be controlled, which activates new moonlighting functions. On the other hand, both pathological conditions stalled in tissue destruction and proliferation [13] can activate a certain moonlighting function at any one time. If this moonlighting function stops tissue destruction or promotes proliferation, it will be considered protective in the first case and pathological in the second, and it will be considered the opposite otherwise. This adds a new layer of complexity in understanding the link between moonlighting proteins and pathology. We try to ease this complexity in the present work, reporting the activation, co-regulation of processes, and alternation between moonlighting functions in disease progression.

## 2. Methods

The moonlighting proteins used in this work are from the MultitaskProtDBII database [5]. This database contains 110 human moonlighting proteins involved in human pathologies which have been analyzed in depth. Protein characteristics have been retrieved from The UniProt Consortium (www.uniprot.org) when necessary, and the information present in the Human Mendelian Inheritance in Man (OMIM, www.omim.org) database [14] and Human Gene Mutation Database (HGMD, www.hgmd.cf.ac.uk) was also used [15].

Extensive analysis of the PubMed bibliography was performed for each of the proteins to study the clinical conditions linked to the canonical and moonlighting functions. We studied the link between the functions and the physiological conditions linked to pathology. These physiological conditions comprise both external and internal factors of the cell, including disease-driver mutations, and are referred to in the text as pathological conditions. The mechanism of action and the moment of action along the disease progression has also been reported when known.

The order in which events are concatenated in wound healing was used to map the activity of canonical and moonlighting functions along the wound-healing cycle. In the case of oncological processes, cancer wound healing was taken as a basis. This sequential aggrupation of canonical and moonlighting functions has served as a guideline for identifying protein functions involved in the same phenotypes, wound healing phases, and the level of damage that requires a different level of repair. The parallelism between wound healing and cancer wound healing was established from the fact that the healing process and cancer progression share a large part of the machinery, with the difference that cancer usually remains stagnant in wound-healing phases requiring a certain degree of cell proliferation [16,17,18,19,20,21].

## 3. Results

### 3.1. Function Relationship with Pathology

In this work, moonlighting proteins linked to human pathologies from the MultiTaskProtDBII database [5] were examined. For this subset of proteins, the role in the pathology and the mechanism of action of their canonical and moonlighting functions was studied. Two classifications were then established from the published experiments elsewhere performed on these proteins: the first, based on the relationship between function and pathology (Section 1), and the second, based on the pathology-mediated relationship between protein functions (Section 2).

#### 3.1.1. Canonical Functions Are the Cause but Moonlighting Functions Are the Effect of the Pathology to a Greater Extent

**Figure 1:**
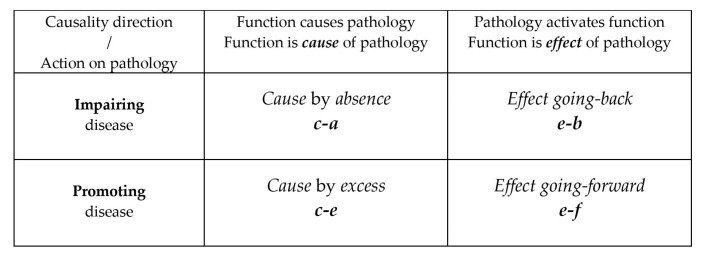
Double classification of protein functions by their role in pathology.

Our first classification describes protein functions according to their role in the pathology using a double classification: (1) by function activation as a *cause* or as an *effect* of the pathology (*cause* vs. *effect* classification), and (2) by whether the function hinders or favors the progression of the pathology with a new symptom onset (impairing vs promoting classification). From this first double classification, four distinct roles in pathology are obtained. They are summarized in Figure 1.

Figure 1: The multiple functions of moonlighting proteins are classified by their role in pathology. Functions are classified both by their action on the pathology and by their causality relation with the pathology, as a *cause* of the pathology or as being activated by the pathology.

- Class 1—The pathology is *caused* by the *absence* of the function (hereinafter summarized as *c-a*).
- Class 2—The pathology is *caused* by an *excess* of function (hereinafter summarized as ***c***-***e***).
- Class 3—The activation of the function is an *effect* of the pathology. The function tries to reverse the pathological condition that triggered it (and its consequences), *back* to the previous non-pathological state (henceforth summarized as *e*-*b*).
- Class 4—The activation of the function is an *effect* of the pathology that requires a more systemic response. The function contributes to the onset of new symptoms *going forward* in the pathological course (hereinafter summarized as ***e***-***f***).

**Figure 2:**
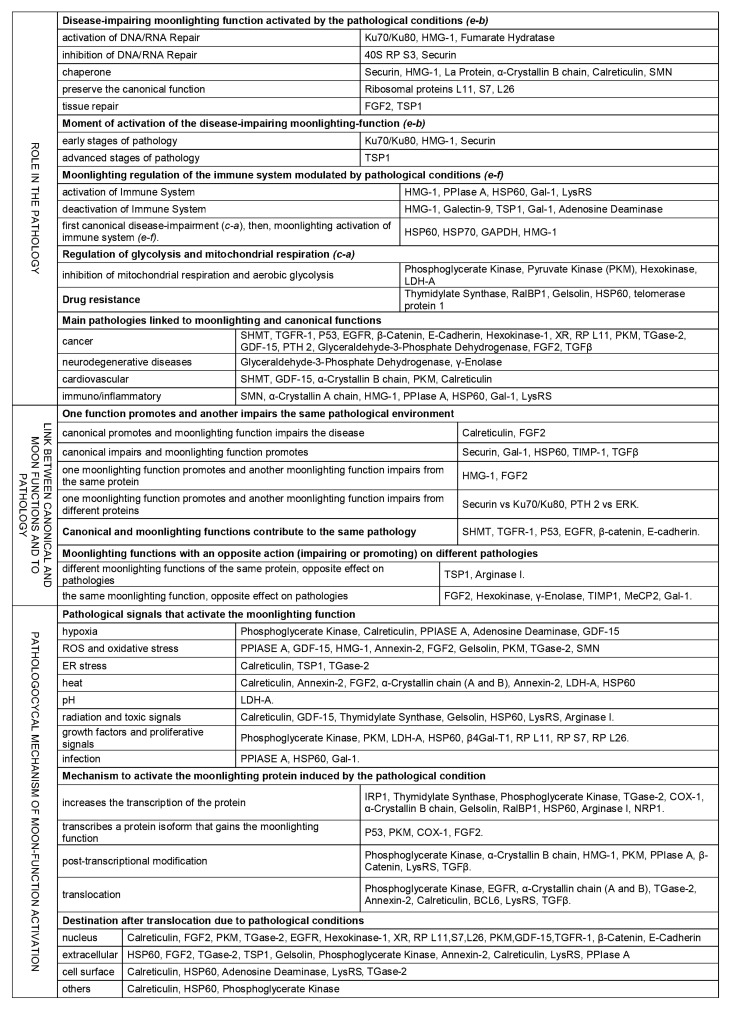
Classification of moonlighting proteins in relation to pathology.

With this double classification we obtain the functions that are the *cause* or *effect* of the pathologies. From the *cause* class, we discriminate by the functions that are *caused* by *absence* (*c-a*) or by *excess* (*c-e*), and from the *effect* class, the functions that after their activation due to pathology, try to *go back* (*e-b*) or *go forward* in the disease (*e-f*). Sees Figure 1 and Figure 2 and Table S1.

From this classification we can state that the role of canonical functions in disease tends to be *cause* (80% of the pathology-related canonical functions found were classified as a *cause*), and mainly *cause* by *absence* (73% *absence* vs. 27% *excess*). This protein *absence* is due to, for example, mutations, inhibitory factors, or a strong abnormal demand that cannot be satisfied. Examples of *cause* by *absence* (*c-a*) are Anion Exchanger 1 in Spherocytosis [22]; Fumarate Hydratase in Fumarase Deficiency Disease (FHD, OMIM 606812); Securin in cancer [23]; Methyl-CpG-Binding Protein 2 in mental retardation (MR, OMIM 300055); or PKM in cardiovascular disease [24]. Some canonical functions are however *cause* by *excess* (*c-e*), such as the excess of Telomerase Component 1 (TEP1), the Lactate Dehydrogenase (LDH-A) excess, and MDM2 excess in cancer [25,26,27]; Hexokinase I in Hyperinsulinism [28]; the HSP90α excess in Huntington disease [29]; or the Nitric Oxide Synthase excess in Parkinson [30]. The moonlighting functions linked to pathology tend to be an *effect* of the pathology (87% of the proteins with moonlighting functions related with pathology were classified as *effect*). Most of them present activation only under the pathological condition that defines their role in the pathology. The protein functions used as examples are detailed in Supplementary Table S3.

Figure 2: Pathology-related moonlighting proteins can be classified by the link among their functions and the link to the pathology. The pathological conditions activate some moonlighting functions which try to regulate these pathological conditions. Some of them are activated at early stages and some at advanced ones, some affect drug activity, some act at the cellular level and other are more systemic, and mono but especially multi-genic diseases are affected, sometimes by different functions of the same moonlighting protein. The moonlighting functions not only regulate pathological conditions but also the effect of other functions when this effect turns pathological. The pathological triggers that activate moonlighting functions are common among multiple proteins (hypoxia, ROS, ER stress, heat, PH, infection, toxins or growth factor) as well as the molecular mechanisms that replace the canonical by the moonlighting function (i.e., translocation, post-transcriptional modifications, an increase in expression, alternative-isoform expression with extra function). The moonlighting proteins cited in the main text have been classified in this figure. Details and bibliographic references about each classification are disclosed in the main text.

#### 3.1.2. Preventive (*e–b*) Functions Are Predominant at Early Stages and Symptomatic (*e–f*) Functions at Later Stages of the Pathology

Both canonical and moonlighting functions can be protective against pathological conditions. The preventive action can be carried out constitutively, when the pathological conditions are not present, as with Heat Shock Proteins [31], La Protein protecting RNA from 3′-end digestion [32], Glutathione Peroxidase-4 [33], or Excision Repair 2 [34]. However, some preventive functions appear just when the pathological conditions become present, such as Sodium/Nucleoside Cotransporter 1 inhibiting tumor growth [35], Cytochrome C causing the apoptosis of damaged cells [36], or Fumarate Hydratase protecting cells from DNA damage when the damage translocates the protein to the nucleus [37]. The constitutive protection is mainly carried out by canonical functions, and the pathology-activated protection is carried out by moonlighting functions. This moonlighting protection is usually activated by the pathological phenotype they are trying to reverse. These moonlighting *going-back* functions are mainly activated in the early stages of the pathology, but can also be present in more advanced pathological conditions, trying to hinder the progression towards even worse stages. For example, the Survival Motor Neuron Protein (SMN) exerts its protective moonlighting function in the early stages of stress-associated pathologies (*e-b*) such as spinal muscular atrophy or sclerosis [38]. However, Thrombospondin-1 (TSP1) exerts its protective moonlighting function a few steps before cancer remission in well-defined tumors (*e-b*) [39]. TSP1 expression is in fact a marker of patient survival [39]. The moonlighting functions that contribute to the disease progression (*e-f*) are usually observed in advanced stages of the disease or close to them. An example would be β 4-Galactosyltransferase 1 (β4Gal-T1) in response to the estrogenic signal in cancer [40]. At earlier stages of the pathology, the ‘*going back*’ function can revert the pathological condition if the damage is low enough (*e-b*). However, when reversion is not possible, new moonlighting functions can carry out a more systemic response (*e-f*), unfortunately contributing to the disease progression and symptom onset. The protein functions used as examples are detailed in Supplementary Table S3.

### 3.2. Dependence among the Multiple Functions of the Proteins in Disease

In our second classification of moonlighting proteins, we classified the relationship among multiple protein functions when this relationship was found. This protein dependence can be between canonical and moonlighting functions; among moonlighting functions; and between functions from the same or different proteins. In relationships between canonical and moonlighting functions, the canonical function was usually found to be linked to the healthy state (*c-a*, *c-e*), and the moonlighting function to be linked to the pathological one (*e-b*, *e-f*). When the dependence is between two moonlighting functions, both are usually activated in pathological conditions (*e-b*, *e-f*), and the variations in these conditions modulate the activation of each function.

The dependence between moonlighting-protein functions is classified then by its relationship with disease. In Figure 2, multipurpose proteins are classified into three categories by the reciprocal effect of function on pathology and pathology on function, linking canonical and moonlighting functions along the way. In Table S1 of the Supplementary Material, for each pathology-related moonlighting protein, the following are shown: (1) the pathologies linked to its canonical and moonlighting functions; and (2) the role of these functions in the pathology (using the *c-a*, *c-e*, *e-b*, *e-f* classification). The proteins described in Figure 2 are a selection of the proteins in Table S1 used in the document as an example.

#### 3.2.1. Mechanisms for Function Activation Mediated by Pathology

##### Function Activation: Multiple Functions of the Same Protein Are Linked to the Same Pathology, but They Are Activated at Different Stages

Some proteins have multiple functions involved in the same pathology, and these functions are progressively activated by the new conditions of the subsequent stages. This is especially common in cancer-related moonlighting proteins such as serine hydroxymethyltransferase (SHMT), TGF-β Receptor type-1 (TGFR1), cellular tumor antigen p53, epidermal growth factor receptor (EGFR), β-Catenin, or E-Cadherin. Their different moonlighting functions lead to different symptoms at each new pathological stage, contributing to the disease progression (*e-f*) [41,42,43,44,45,46].

##### Function Activation: The Moonlighting Function Is Activated by Changes in Cellular Localization Mediated by Pathological Conditions

The cellular localization in many cases determines which function is finally activated in these proteins with extra functions. This translocation is usually due to pathological conditions. We show some examples in this paper. Under pathological ER stress, calreticulin (CRT) is translocated and even extracellularly released to carry out its moonlighting functions [47]. Reduced exogenous high mobility group protein B1 increases autophagy (necrolytic state), but oxidized HMG-1 increases apoptosis in a localization-dependent mode [48]. The mutation of cysteine 106 of HMG-1 promotes the cytosolic localization and subsequent sustained autophagy [48]. The accumulation of HMG-1 at sites of oxidative DNA damage can also lead to DNA repair (*e-b*) [48]. Peptidyl-prolyl cis-trans isomerase A (PPIase A) can be secreted into the extracellular environment in various cell types due to inflammatory stimuli such as infection, hypoxia, and oxidative stress to perform its pro-inflammatory moonlighting function (*e-f*) [49]. Lysine-tRNA ligase (LysRS) also has an extracellular pro-inflammatory moonlighting function (*e-f*) [50] and adenosine deaminase at the cell surface reduces extracellular adenosine levels [51]. Galectin-1 (Gal-1) is also extracellularly released during infection or inflammation, but the secreted extracellular Gal-1 is described as a strong immunosuppressor (*e-b*), unlike the intracellular Gal-1 [52]. In cases of neurodegenerative amyotrophic lateral sclerosis, Gal-1 accumulates in the neurofilamentous lesions and shows a neuroprotective effect (*e-b*) [53]. Gal-1 presents a similar behaviour in Ischemia (*e-b*) [53]. Numerous proteins migrate to the nucleus in cancer to exert their moonlighting function as a transcription factor or that are involved in repair, including, among others: Hexokinase-2 [54]; L-Xylulose Reductase (XR) [55]; 60S Ribosomal Protein L11 [56]; Pyruvate Kinase PKM2 [57], Protein-Glutamine γ-Glutamyltransferase 2 (TG2) [58]; Growth/Differentiation Factor 15 (GDF-15) [59]; TGF-β Receptor type-1 (TGFR1) [60]; Epidermal Growth Factor Receptor (EGFR) [61]; β-Catenin [62]; or E-Cadherin [63].

Different localizations can activate different moonlighting functions of the same protein causing very different symptoms, even opposite ones, ending in different pathologies as a result (*e-f*). Arginase I expression is augmented in response to exposures to environmental air pollutants promoting asthma [64], but Myeloid-Derived Suppressor Cells (MDSCs) producing high levels of Arginase I block T cell function in cancer, chronic infections, and trauma patients [65].

##### Function Activation: The Moonlighting Function Is Activated by Transcriptional and Post-Transcriptional Changes Mediated by Pathological Conditions

The pathological microenvironment increases the expression of some isoforms incorporating moonlighting functions and activates them by means of their post-transcriptional regulation. There are multiple examples of this post-transcriptional activation of the moonlighting function by pathological conditions: High Mobility Group Protein B1 is post-transcriptionally modulated by ROS [48]; β 4-Galactosyltransferase 1 is post-transcriptionally modulated by estrogens [66]; Ribosomal Proteins L11, S7 and L26 are post-transcriptional modulated by serum starvation [56,67]; Adenosine deaminase is post-transcriptional modulated by hypoxia [68]; and Protein-Glutamine γ-Glutamyltransferase 2 is post-transcriptionally modulated by a huge amount of pathological stimulus [58]. In some cases, the moonlighting function is also activated by the transcriptional changes caused by pathological conditions. The pathological environment establishes the isoform (usually by RNA splicing) of the gene to be transcribed, translating a different protein with extra functions. These alternative isoforms will gain extra moonlighting functions without losing the original function, thus becoming a new moonlighting protein. The main isoform present in healthy and homeostatic conditions may or may not be moonlighting. Fibroblast Growth Factor 2 (FGF2) is synthesized by cells as high or low molecular weight isoform from a single mRNA, translated respectively from CUG or AUG start sites depending on the conditions. A variety of stress stimuli, including oxidative stress and heat shock, have been reported to favor translation from CUG sites accumulating Hi-FGF-2 isoforms. The CUG-initiated or Hi-FGF-2 isoforms are localized in the nucleus and are responsible for the intracrine effect, whereas the AUG-initiated or Lo-FGF-2 form is mostly cytosolic and is responsible for the paracrine and autocrine effects. Lo-FGF-2 is a moonlighting protein that promotes endothelial cell migration and angiogenesis, while Hi-FGF-2 inhibits it [69]. Pyruvate Kinase PKM2 is another example. High glycolysis levels induce PKM alternative splicing resulting in a new moonlighting protein. In turn, mitochondrial reactive oxygen species promote dimerization of this PKM alternative isoform and enable its nuclear translocation [24]. The dimeric PKM alternative isoform is also released into the circulation of cancer patients, promoting angiogenesis [70]. PKM2 moonlighting functions linked to pathology are performed by the dimeric form of PKM2, whereas the canonical enzymatic activity is performed by the tetrameric form [70]. Thus, pathological conditions, such as growth signals, first promote the transcription of the *pkm* alternative isoform and thereafter its dimerization. Nonetheless, the canonical function is still present in the alternative isoform [70]. In cancer cells, the alternative splicing of *pkm* RNA replaces the usual isoform [70]. At least twelve p53 protein isoforms have been described to be encoded by nine p53 mRNAs [71], many of them linked to extra moonlighting functions exerted only in pathological conditions [72]. Function Activation: The Same Moonlighting Function Acts as a Going Back Function in One Disease and as a Going Forward Function in Another Disease

The functions of the same protein sometimes try to reverse some pathologies (*e-b*), but contribute to the pathologies of other ones (*e-f*). In some cases, it is the same protein but two of its functions, each one with an opposite role in each disease. In other cases, the same moonlighting function plays a *back* or *forward* opposite role depending on the disease. Thrombospondin-1 (TSP1) has different moonlighting functions with opposite roles in different pathologies. TSP1 circulates in response to a High-Fat Diet, and the moonlighting function may induce insulin resistance (*e-f*) [73]. However, TSP1 also participates in tumor remission in multiple ways (*e-b*) [39]. The same moonlighting function of Galectin-1 has an opposite role depending on the pathology. Its moonlighting function stops the immune response in autoimmune diseases or asthma (*e-b*) [74], but also stops immune response in cancer (*e-f*) [75]. Depending on the repair phase in which the disease is stalled, the same protein function can be clinically seen as protective or pathological, depending on whether it breaks the stalling or contributes to it.

#### 3.2.2. Types of Relationships among Canonical and Moonlighting Functions

##### Moon-Canonical Link: The Moonlighting Function Tries to Compensate for an Excess of the Canonical Function of the Same Protein

In cases where the pathology is linked to the *excess* of the canonical function of the protein (*c-e*), the moonlighting function tries to compensate for this *excess* by going *back* to the healthy state (*e-b*). For example, calreticulin, whose canonical function promotes cell stress via calcium release, tries to compensate for pathological cell stress through its moonlighting functions: as a chaperone [47], as an inhibitor of the STAT3 pathway [76], and finally as an “eat me” signal. It elicits the later phagocytosis of already dysfunctional and dying cells due to the accumulated stress [77]. In this way we pass from a *going back* moonlighting function to a *going forward* one.

##### Moon-Moon Link: The Moonlighting Going Back Function Tries to Stop a Moonlighting Going Forward Function of the Same Protein

In some cases, the progression of the pathology depends on the balance between *going-back* and *going forward* functions of the protein. In these cases, a moonlighting function tries to compensate (*e-b*) for the symptoms caused by the other moonlighting function (*e-f*) of the same protein. High Mobility Group Protein B1 is an example. The mammalian immune system discriminates between two modes of cell death: necrosis, which often results in inflammation, and apoptosis, which tends to be anti-inflammatory and promote immune tolerance. This switch between the two responses may depend on the HMG-1 moonlighting function finally activated, which depends in turn on the different pathological environment (necrolytic or apoptotic). In pathological conditions, the pro-inflammatory moonlighting function is activated, but the oxidation of some amino acid residues by ROS moves HMG-1 from the pro-inflammatory to the anti-inflammatory activity [48]. In this way, the switch from one moonlighting function to the other can lead to a different pathology, to an anti-inflammatory apoptosis or to a pro-inflammatory necrosis [78].

##### Moon-Moon Link: The Moonlighting Going Back Function Tries to Stop a Moonlighting Going Forward Function of a Different Protein

There is also compensation between moonlighting functions exerted by different proteins. In these cases, the moonlighting function of a protein tries to compensate/delay/diminish (*e-b*) the symptoms (*e-f*) caused by the moonlighting function of another protein. For example, several moonlighting proteins have moonlighting functions that activate the ERK pathway (*e-f*) [79], promoting the creation of stroma-invasive niches. In contrast, the moonlighting function of mitochondrial Peptidyl-tRNA Hydrolase 2 (PTH 2) impairs metastasis by inhibiting ERK (*e-b*) [80]. This cross-talk between moonlighting functions of different proteins reveals the complexity in the regulation of processes that moonlighting proteins consolidate under pathological conditions.

##### Moon-Canonical Link: The Moonlighting Going Forward Function (e–f) Is Activated when the Preventive Function Fails (c–a)

Some moonlighting proteins have a protective role against pathology in their canonical function (*c-a*) but contribute to the disease progression when its moonlighting function is activated (*e-f*). Initially, the protein prevents the pathology but later promotes it. For example, Metalloproteinase Inhibitor 1 (TIMP-1) inhibits Interstitial collagenase (MMP-1) canonically, but its moonlighting function activates cell proliferation and survival in cancer [81,82]. Likewise, TIMP-1 is an MMP-inhibitor at the cancer periphery but is involved in tumor-induced angiogenesis in the pericytes [83]. Protein TGFβ Receptor 1 is also initially a tumor suppressor, since its canonical function inhibits cell proliferation and induces apoptosis, but later, the TGFβ Receptor 1 moonlighting function leads to tumor progression. The TGFβ moonlighting function requires translocation and post-transcriptional modifications caused by the environment of these later stages [72].

In several multipurpose proteins, the moonlighting function facilitates the immune response against the pathological condition that the canonical function was initially trying to reverse. That is, the canonical function first tries to prevent the pathological condition, but being unable to, the protein becomes an activator of the immune system, trying to repair the damage at a more systemic level, usually involving the onset of new symptoms. The 60 kDa heat shock protein (HSP60) is a mitochondrial chaperone (canonical function). Upon long exposure to stress, HSP60 is also found in the cytosol, cell surface, extracellular space and biological fluids. HSP60 activates innate and adaptive immune responses (moonlighting functions) and can function as an endogenous danger signal to the immune system [84]. The more intense the initial stress exposure is, the higher its transcription will be, and the greater the posterior immune response due to the moonlighting function will be [85].

##### Moon-Canonical Link: Moonlighting Going Back Function Is Activated to Prevent Canonical-Function Failure in Adverse Conditions

A different kind of moonlighting protein exerts their preventive moonlighting function (*e-b*) by trying to preserve its canonical function in pathological conditions. Unlike the previous case, the pathological conditions are not caused by the canonical function failure, but the canonical function needs to be preserved under these pathological conditions. The moonlighting function then tries to make this canonical function activity possible despite the adverse conditions. Ribosomal proteins L11, S7, and L26 are an example. The 60S ribosomal protein L11 plays a dual role as either a component of the 60S ribosomal subunit for protein synthesis under favorable growth conditions, or as a component of the HDM2–P53 pathway, impairing cell cycle progression, under growth-inhibitory conditions (e.g., by serum starvation). The moonlighting function is activated after protein translocation to the nucleoplasm [86]. When the ribosomal-biogenesis integrity is threatened, the more intense the ribosomal biogenesis, the higher the ribosomal-proteins transcription and the greater the proliferation inhibition [86]. The moonlighting function described in the previous section was an *e-f* subtype, whereas in the current section an *e-b* subtype is described, but in both cases, the levels of moonlighting protein transcription are determined by the needs of canonical-function. In neither of the two cases does the pathological condition up-regulate the protein synthesis, and the moonlighting response remains proportional to the initial canonical-function demand. The initial canonical-function demand sets the expression levels of these moonlighting proteins and the moonlighting function is just postranscriptionally regulated.

### 3.3. Moonlighting Proteins in Wound-Healing, Cancer-Wound-Healing and Mesenchymal to Epithelial Transition (EMT)

As we have previously seen, “*going-forward*” moonlighting functions usually involve a systemic response, such as the activation of the immune response or the formation of niches for tissue regeneration. In both cases, this systemic response attempts to reach a final functional state as a result of the repair process; however, this functional state is not always achieved. The “*going-forward*” moonlighting functions often lead to inflammatory diseases or tumor progression. As summarized in Table S2, many moonlighting proteins contribute to the immune-led epithelial-mesenchymal transition (48% of the pathology-related proteins). As we have also seen in previous examples, some protein functions—canonical and moonlighting, from the same and different proteins—try to stop tumor development at the unicellular level, but their failure to re-establish the internal balance prompts the activation of more systemic measures, activating the “*going-forward*” moonlighting functions. If the systemic measures imply immunological intervention, a specific program, called “wound healing”, is launched and carried out until the end. This immuno-EMT process involves important alterations, such as changes in the type of immuno-cells infiltrated in the tissue, the passage from aberrant-cell destruction to proliferation, or the passage from an epithelial to a mesenchymal cell morphology, as well as the step back to epithelial morphology when trying to close the wound-healing cycle and re-establish tissue activity. In light of our previous findings, we evaluated the extent to which the moonlighting proteins linked to pathology could be involved in these wound-healing and cancer-wound-healing processes.

The wound healing process follows a sequence of phases partially overlapped that goes from the destruction to the proliferation of the tissue with the aim of remodeling it and repairs supposed previous damage. In pathologies, the destructive or proliferative part becomes stalled [21]. Tissue destruction is dominated by apoptosis and inflammation calling, while tissue proliferation ranges from niche creation, with stem cell transformation, ERK pathway activation, and stem cell expansion, to proliferation of the affected tissue, with the activation of the mTOR pathway, the proliferation of the stroma, the end of inflammation and, finally, the completion of the wound healing cycle [16,17,18,19,20]. Between successive cycles of wound healing, there is the activity phase, in which the tissue is stressed. Multiple moonlighting proteins are involved in each of these phases, as reflected in Table 1.

Table 1: The different moonlighting functions of different proteins are essential to carry out the sequential phases of the wound-healing process. These sequential phases are divided into the destructive and constructive part of the tissue remodeling carried out by the wound-healing process. Each occurrence of the same protein (in the table) represents a different function of the protein. Consequently, the moonlighting proteins linked to different processes perform a different function in each of these processes. This mutual exclusivity between function-phase pairs is key in the transitions between wound-healing phases. The moonlighting functions that reinforce a specific wound-healing phase produce an indirect inhibitory effect on the previous and subsequent phases. These negative regulations of the wound healing phases are not included in the table. Only positive regulations are shown. On the other hand, invasiveness (in the table) is a process specific to the increased response to worsening pathological conditions in cancer.

Some moonlighting functions are not only essential to carry out one wound-healing phase, but also guide the transition from one phase to the next, launching the phenotype of the next phase and inhibiting that of the previous one. Scrib and GDF-15 moonlighting functions stop the ERK pathway to activate the mTOR pathway [134,144]. In this way they stop the stem cells’ expansion and promote re-epithelialization [119]. GDF-15 also stops inflammation to activate the mTOR pathway [149]. As shown in Table 1, many moonlighting proteins change their function from one phase to another, participating in specific processes in each phase. The Wound-Healing proteins TGFBR1 and SMAD link apoptosis [96,97,98], cell transformation into cancer stem cells [111,112,113], and invasiveness [113] through their different functions. Heat Shock Proteins such as HSP70 and HSP60 link their protective role under stress [150,151] with the Wound-Healing inflammation calling [152] and its later suppression [153,154]. The glycolytic proteins PFK1 and Aldolase participate with their moonlighting functions in the invasiveness (by PFK1) and the mTOR pathway (by Aldolase). On the contrary, the glycogenic protein FBP1 inhibits the ERK pathway. Thus, these moonlighting proteins are ensuring the energy availability in the main tissue-remodeling processes [131].

In addition to the transition between wound-healing phases, moonlighting functions are also involved in increasing the Wound-Healing response, making this response to damage more aggressive and extensive. PKM2 changes from aerobic to anaerobic respiration [131]. Scrib [144], LARS1 [155] and GDF-15 [149] contribute to the switch from an inflammatory response to a proliferative one via mTOR-pathway activation. The β-catenin translocation acts in a similar way [141]; both cases lead to tumor initiation with the imbalance between destruction and proliferation in tissue remodeling [141,156]. Invasiveness is promoted by multiple moonlighting proteins; for example, the tumor becomes invasive through a specific moonlighting function of HSP90 [126]. The activation of the Rho-Rock pathway by a specific moonlighting function of TG2 also provides invasive properties to cancer stem cells [157,158]. Invasiveness is key in worsening cancer wound healing and moonlighting proteins are key in invasiveness initiation and promotion (Table 1).

## 4. Discussion

In the present work we have shown a detailed literature analysis of 110 human moonlighting proteins whose functions are linked to pathology from the MultitaskProtDBII database at http://wallace.uab.es/multitaskII/ [5]. The 110 proteins analyzed are the most studied human moonlighting proteins and with the largest available bibliography. The multiple functions of these proteins have then been classified in relation to pathology (Figure 2 and Table S1), in relation to other multipurpose functions (Figure 2), and in relation to EMT (Table S2) as well as to the main processes involved in wound healing (Table 1).

We have seen that the canonical functions linked to pathology act mainly as a causal vector of the pathology (*cause*), and the moonlighting functions linked to pathology are activated mainly as an effect of the pathology (*effect*). This different relationship of canonical and moonlighting functions with the pathology could have an evolutionary purpose. If the pathology activates the moonlighting function to respond to the damage, the moonlighting function is expected to be the most recently acquired. If the canonical function is involved in key ancestral functions, such as primary metabolism, its mutation or inhibition is more deleterious and is a possible cause of the pathology. The neomorphic functions described by [12] are novel functions that evolved from the adaptation of existing functions, reusing pre-existing proteins and induced by mutations. Although it is difficult to decipher how both functions evolve, a single polypeptide with functions adapted to multiple physiological states arising from clinical conditions could represent an advantage for the cell and the organism. The main advantage is that even though a single isoform is expressed and transcriptionally regulated, all functions of the protein are covered by this single expression. The final switch between functions will depend on the post-translational regulation of the protein. This fact also allows mutual exclusivity between functions, which is especially useful if the two functions of the same protein are determinant for alternating phenotypes. In addition, after the evolutionary emergence of the moonlighting protein, the organism has a single protein with specific responses both to normotony and to high levels of damage. On the other hand, although moonlighting proteins could increase pleiotropy and, therefore, dilute the efficiency of selective pressure on genes, moonlighting proteins would explain how a relatively low number of proteins can perform the large number of functions required to sustain life, especially in response to damage.

Under non-pathological conditions, moonlighting proteins do not show an evident relationship between their different functions. What we describe in this paper was initially unexpected. We found that under pathological conditions, a very significant number of human moonlighting proteins have a relationship between their canonical and moonlighting functions, between the moonlighting functions of the same protein, as well as with moonlighting functions of other proteins. Indeed, it turned out that the pathological conditions regulate their relative activity. This should be less surprising since disease databases like Malacards (www.malacards.org) [159] or OpenTargets (https://www.targetvalidation.org) [160], show (a) that most diseases are multigenic, and (b) that most key proteins are related to many different diseases. A gene-disease network analysis also discloses functional modules involved in multiple human diseases [160]. In addition, these diseases are mostly led by the immune system and linked to the wound healing process. This is something that we also observed when we studied the pathology-related moonlighting proteins (Table 1). As described herein, moonlighting proteins perform the function triggered by the pathologic conditions of each wound-healing phase, contributing as well to the transition between phases. Moonlighting proteins can also increase the level of response, often deepening and spreading the disease. Nevertheless, many of these moonlighting functions are just trying to restore normalcy after the damage, but with a non-successful outcome. Therefore, this worsening could be a clinical interpretation of the *going-forward* moonlighting functions. The accumulated damage seems to be the determining factor in the failure of the preventive functions and the activation of these *going-forward* functions.

As part of the wound healing, cancer wound healing, and EMT, moonlighting proteins are directly involved in autoimmune, inflammatory, and cancerous diseases, as well as in the transition between these pathologies. We found quite a few pathology-related moonlighting functions that appear exclusively in the wound healing process, and we included some of the most representative in this work (Table 1). These moonlighting functions are synchronized to carry out the destructive and constructive processes that comprise the wound healing. When these processes are intensified, or even stalled, new moonlighting functions of different proteins can be activated. Some of these functions will try to stop the extension of the destructive and constructive processes, whereas others will deepen those transformative processes.

The role of moonlighting functions in disease complicates the rational design of therapies to a greater extent. Treating the canonical function of a multifunctional protein may not be enough to cure the disease, considering the high activity of moonlighting functions in pathology, especially in advanced stages. This highlights the usefulness of designing methods to identify moonlighting proteins experimentally or in silico [5,8,161,162,163,164,165,166,167,168,169,170,171]. Active sites residues which are involved in their moonlighting functions are very relevant during drug designing against these proteins targeting specific moonlighting functions [172]. For instance, different Fumarase active sites are linked to different pathologies, requiring site-specific targeting (Uniprot:P07954). However, this would not be enough. The participation of moonlighting proteins in the repair processes launched throughout the course of the disease makes it necessary to map the new functions to the wound healing. This will help to predict, at the time of treatment, the current and future activated function of the protein which is necessary to avoid first- and second-line resistance. Drug design must be adapted to moonlighting functions both in time (moment of function activation) and space (protein structure).

In future works, we will continue with the collection and manual curing of proteins with moonlighting functions, delving into their role in the two dimensions of the wound healing approach: (1) in the transitions between phases throughout the wound healing cycle; and (2) in the alterations of the wound healing cycle when the remodeling response intensifies as the disease progresses. All of this is in light of the current results and is motivated by the involvement of the wound healing process in pathology and the significant involvement of moonlighting proteins in the wound healing process.

## Figures and Tables

**Table 1:** Wound-healing and cancer-wound-healing main processes and their specific moonlighting functions.

TISSUE ACTIVITY		Stress	Stress protection	HSPs [87,88,89], GAPDH [90], TG2 [91], FXIIIA [92], HMGB1 [93], PKM2 [94,95]
WOUND HEALING CYCLE	TISSUE DESTRUCTION	Clearance	Apoptosis (intrinsic)	TGFBR1 [96,97,98], SMAD3 [96,97,98], GAPDH [99], TG2 [100], ESE-1 [101], CRT [102], HMGB1 [103]
			Inflammation (cytotoxic and scavenger)	HSP70 [104,105], HSP60 [106], TG2 [107], ESE-1 [108,109], CRT [110], HMGB1 [103]
	TISSUE CREATION	Niche creation	Stem cells (cell transformation)	TGFBR1 [111,112,113], SMAD3 [111,112,113], E-Cadherin [114], TG2 [115,116], ESE-1 [117], HMGB1 [118]
			ERK pathway activation	TGFBR1 [98], EGFR [119], HSP90 [120], E-Cadherin [121], TG2 [122], PKM2 [123]
			Stem-cell self-renewal (symmetric proliferation)	β-Catenin [124], HMGB1 [125]
			Invasiveness	TGFBR1 [113], SMAD3 [113], HSP90 [120,126], β-Catenin [127], TG2 [128,129], ESE-1 [117], ATF2 [130], FPK1 [131]
		Extra-cellular matrix remodelling	Inflammation termination	HSP70 [104], HSP60 [132], GAPDH [133], HMGB1 [103], GDF-15 [134]
			Fibrosis	SMAD3 [135], TG2 [122,136], FXIIIA [133], ESE-1 [137,138]
			Angiogenesis	PKM2 [70], HMGB1 [139]
		Re-epithelization	Epithelial proliferation	EGFR1 [140], β-Catenin [141], PKM2 [142]
			mTOR pathway activation	ESE-1 [143], Scrib [144], GDF-15 [134], LARS1 [145], Aldolase [131]
			Differentiation (epithelial)	ESE-1 [146]
			Wound healing termination	GAPDH [147], β-Catenin [148]

## Data Availability

The data supporting reported results are available from the corresponding author on request.

## Supplementary Materials

The following supporting information can be downloaded at: https://www.mdpi.com/article/10.3390/cells12020235/s1, Figure S1: Fumarase; Table S1: Causes and effects; Table S2: EMT; Table S3: Functional Details.
